# Cloning, purification and structure determination of the HIV integrase-binding domain of lens epithelium-derived growth factor

**DOI:** 10.1107/S2053230X18001553

**Published:** 2018-02-26

**Authors:** Clare Hannon, Abimael Cruz-Migoni, Olga Platonova, Robin L. Owen, Joanne E. Nettleship, Ami Miller, Stephen B. Carr, Gemma Harris, Terence H. Rabbitts, Simon E. V. Phillips

**Affiliations:** a Weatherall Institute of Molecular Medicine, University of Oxford, John Radcliffe Hospital, Oxford OX3 9DS, England; b West Suffolk Hospital, Hardwick Lane, Bury St Edmunds IP33 2QZ, England; c Diamond Light Source, Rutherford Appleton Laboratory, Didcot OX11 0DE, England; d Oxford Protein Production Facility, Research Complex at Harwell, Rutherford Appleton Laboratory, Didcot OX11 0FA, England; e Division of Structural Biology, Henry Wellcome Building for Genomic Medicine, University of Oxford, Roosevelt Drive, Oxford OX3 7BN, England; f Department of Biochemistry, University of Oxford, South Parks Road, Oxford OX1 3QU, England; g Research Complex at Harwell, Rutherford Appleton Laboratory, Didcot OX11 0FA, England

**Keywords:** HIV integrase-binding domain, lens epithelium-derived growth factor, human immunodeficiency virus, domain swapping

## Abstract

Lens epithelium-derived growth factor (LEDGF)/p75 is the dominant binding partner of HIV-1 integrase in human cells. The crystal structure of the HIV integrase-binding domain (IBD) of LEDGF has been determined in the absence of ligand. IBD was overexpressed in *Escherichia coli*, purified and crystallized by sitting-drop vapour diffusion. X-ray diffraction data were collected at Diamond Light Source to a resolution of 2.05 Å. The crystals belonged to space group *P*2_1_, with eight polypeptide chains in the asymmetric unit arranged as an unusual octamer composed of four domain-swapped IBD dimers. IBD exists as a mixture of monomers and dimers in concentrated solutions, but the dimers are unlikely to be biologically relevant.

## Abstract 2

The HIV integrase-binding domain (IBD) of lens epithelium-derived growth factor has been cloned, purified and crystallized, and its structure has been solved. IBD forms an unusual domain-swapped dimer that assembles into octamers in the crystal.

## 1. Introduction   

Lens epithelium-derived growth factor (LEDGF) is a transcriptional co-activator that was discovered as a binding partner of HIV integrase (Cherepanov *et al.*, 2003 ▸). It was later shown to be essential for the formation of a tripartite complex of MLL (mixed lineage leukaemia; HGNC nomenclature KMT2A) protein, menin (multiple endocrine neoplasia type 1; MEN-1) protein and LEDGF that is implicated in MLL (Cermáková *et al.*, 2014 ▸). Epitope mapping shows that the integrase-binding domain (IBD) of LEDGF is also the part of the protein necessary for MLL/menin binding (Cermáková *et al.*, 2014 ▸). The design of drugs that can interfere with protein–protein interactions (PPIs) involving IBD would be important for therapy in both AIDS and leukaemias.

Drug screens for small molecules, or for macromolecules such as intracellular antibody fragments, require structural data for *in silico* design and/or epitope targeting. The structure of IBD has previously been determined in solution by NMR (Cherepanov, Sun *et al.*, 2005 ▸) and in X-ray crystal structures of complexes of IBD with HIV integrase as a heterodimer (Cherepanov, Ambrosio *et al.*, 2005 ▸) and a tetramer (Hare *et al.*, 2009 ▸), with an HIV integrase homologue (Hare & Cherepanov, 2009 ▸) and in complex with menin and MLL (Huang *et al.*, 2012 ▸). The crystal structure of IBD alone, however, has not been solved in the absence of ligands. In this paper, we report the cloning, overexpression, purification and X-ray crystal structure determination of free IBD.

## 2. Materials and methods   

### 2.1. Macromolecule production   

The sequence encoding the HIV integrase-binding domain (IBD) of LEDGF was PCR-amplified directly from cDNA prepared using an RT-PCR kit with RNA extracted from the T-cell lymphoma cell line VL3-3M2 (Groves *et al.*, 1995 ▸). The purified DNA fragment (372 bp) was inserted into the NotI and EcoRI sites of pRK172 vector, encoding an N-terminal His tag, a TEV protease cleavage site and a 24-amino-acid linker ending with methionine preceding the initial Glu345 of the IBD. Positive clones were confirmed by colony PCR, and plasmid DNA was isolated and purified using a QIAquick plasmid kit (Qiagen, Crawley, England). The correct sequence was confirmed and the plasmid DNA was transformed into *Escherichia coli* B834 (DE3) cells. For the production of IBD protein, a 50 ml flask containing 10 ml Power Broth Medium (Molecular Dimensions) supplemented with 50 µg ml^−1^ carbenicillin was inoculated with a single colony of the transformed *E. coli* cells and grown overnight at 310 K in a shaking incubator at 225 rev min^−1^. 8 ml of this culture was used to inoculate 2 l flasks each containing 0.5 l Power Broth Medium (Molecular Dimensions) supplemented with 50 µg ml^−1^ carbenicillin. Growth was carried out at 310 K with vigorous aeration until an OD_600_ of 0.6 was attained and the cultures were induced with isopropyl β-d-1-thiogalactopyranoside (IPTG) at a final concentration of 0.5 m*M*. The temperature was reduced to 298 K and the culture was incubated for an additional 18 h.

The cells were harvested by centrifugation at 5000*g* for 10 min at 277 K and lysed using a cell disrupter (Constant Systems Ltd, UK) at 186 MPa in lysis buffer (50 m*M* Tris pH 7.5, 500 m*M* NaCl, 30 m*M* imidazole, 0.2% Tween) supplemented with protease inhibitor (cOmplete EDTA-free tablets, Roche Life Science, UK) and DNase I. Cell debris was removed by centrifugation at 50 000*g* for 50 min at 227 K (Beckman Coulter Avanti J-26 XP with JA 25.50 rotor) and the supernatant was collected. The protein was purified using an automated IMAC–SEC process on an ÄKTAxpress system (GE Healthcare). Firstly, the supernatant was loaded onto a 1 ml HisTrap FF column (GE Healthcare) and washed using a buffer consisting of 50 m*M* Tris pH 7.5, 500 m*M* NaCl, 30 m*M* imidazole. Elution in a buffer consisting of 50 m*M* Tris pH 7.5, 500 m*M* NaCl, 500 m*M* imidazole was followed by direct injection onto a HiLoad Superdex 75 pg 16/60 column (GE Healthcare, UK) and elution in 20 m*M* Tris pH 7.5, 200 m*M* NaCl, 1 m*M* TCEP; fractions were collected and analysed by SDS–PAGE. Fractions containing the IBD protein were concentrated to 2 ml using an Amicon Ultra-15 concentration device with a 3 kDa molecular-weight cutoff before the addition of a 0.1× volume of TEV protease (1 mg ml^−1^) (a kind gift from the Membrane Protein Laboratory, Diamond Light Source, UK). The sample was incubated overnight at 277 K to allow proteolytic cleavage of the His tag. The protein was further purified by passing it through a HisTrap FF column (reverse IMAC) to remove the tag and His-tagged TEV protease from the IBD protein. Finally, the IBD protein was concentrated to 6.5 mg ml^−1^ for crystallization using an Amicon Ultra-15 centrifugal concentrator with a 3 kDa molecular-weight cutoff. The purity was estimated to be greater than 95% (as determined by SDS–PAGE). Approximately 0.1 mg pure protein was obtained from 1 l of culture.

The protein was analysed by intact protein mass spectrometry (Nettleship *et al.*, 2008 ▸) and its molecular weight was found to be 11 882 Da, compared with the calculated value of 11 881.66 Da based on its sequence. During purification the His-tagged IBD protein eluted from the HiLoad Superdex 75 pg 16/60 column at 78.05 ml, suggesting that it is a monomer in solution when compared with calibration standards (GE Healthcare).

In the preparation of IBD for analytical centrifugation, pRK172-His-TEV-IBD was transformed into *E. coli* C41 (DE3) cells. A single colony was grown overnight in 80 ml LB containing 100 mg ml^−1^ ampicillin at 37°C and 225 rev min^−1^. 8 ml of the overnight seed culture was used to inoculate 8 × 1 l LB containing 100 mg ml^−1^ ampicillin. The cells were grown at 30°C at 225 rev min^−1^ until an OD_600_ of 0.8 was reached. Protein expression was induced by the addition of 0.1 m*M* IPTG and the cells were incubated at 18°C and 225 rev min^−1^ for a further 3 h. The cells were harvested by centrifugation and the pellets were resuspended in lysis buffer (50 m*M* Tris pH 7.5, 500 m*M* NaCl, 0.2% Tween 20, 20 m*M* imidazole) containing EDTA-free protease-inhibitor cocktail tablets (Roche, Germany), DNase I and 1 m*M* MgSO_4_. The cells were lysed using a cell disruptor (Constant Systems Ltd, UK) at 172 MPa and 4°C and the sample was clarified by centrifugation at 23 000 rev min^−1^ for 1 h. The cell lysate was incubated with Ni–NTA agarose (Qiagen, UK) for 2 h at 4°C. The beads were applied onto a gravity-flow column and were washed with 50 m*M* Tris pH 7.4, 500 m*M* NaCl and 20 m*M* imidazole. Soluble His-TEV-IBD was eluted with 30 ml 50 m*M* Tris pH 7.4, 500 m*M* NaCl and 300 m*M* imidazole. His-TEV protease was added to the eluate at a concentration of 17.5 µg ml^−1^ and the mixture was dialysed against 20 m*M* Tris pH 7.5, 200 m*M* NaCl overnight at 4°C. To remove the cleaved His-TEV, uncleaved His-TEV-IBD and His-TEV protease, the sample was incubated with Ni–NTA beads for 1 h at room temperature. The beads were again applied onto a gravity-flow column and the flowthrough was collected. The IBD sample was concentrated to 2 ml and was further purified by gel filtration using a Superdex 75 16/600 column (GE Healthcare, UK) with 20 m*M* Tris pH 7.5, 200 m*M* NaCl, 1 m*M* TCEP. Protein-containing fractions were pooled and concentrated for analytical ultracentrifugation. Approximately 1.5 ml pure protein was obtained from 1 l of culture.

The oligomeric state of the protein at higher concentrations was analysed by analytical ultracentrifugation (AUC). For characterization of the IBD sample, sedimentation-velocity scans were recorded for a twofold protein-dilution series, starting from 6.5 mg ml^−1^. All AUC experiments were performed at 50 000 rev min^−1^ using a Beckman XL-I analytical ultracentrifuge with an An-50 Ti rotor at 20°C. Data were recorded using the absorbance (at 280 nm) and interference optical detection systems. The density and viscosity of the buffer was measured experimentally using a DMA 5000M densitometer equipped with a Lovis 200ME viscometer module. The partial specific volume of the protein construct was calculated using *SEDFIT* (Schuck, 2000 ▸) from the amino-acid sequence. Data were processed using *SEDFIT*, fitting to the *c*(*s*) model. Figures were made using *GUSSI* (Brautigam, 2015 ▸).

Cloning and protein-purification information is summarized in Table 1 ▸.

### 2.2. Crystallization   

Crystallization screening experiments were performed by sitting-drop vapour diffusion using a Cartesian MicroSys crystallization robot (Digilab Ltd, Huntingdon, England) followed by incubation at 294 K (Walter *et al.*, 2005 ▸). The protein concentration was calibrated from the analytical ultracentrifugation results. Crystals were observed after 24 h in a number of crystallization drops, with the best crystals being obtained using the conditions shown in Table 2 ▸.

### 2.3. Data collection and processing   

Crystals were harvested and transferred to a 2:1 mixture of crystallization buffer and glycerol as a cryoprotectant for a few seconds before flash-cooling in liquid nitrogen. Diffraction data were collected from a single crystal with dimensions of 25 × 25 × 90 µm at 100 K using a wavelength of 0.9778 Å on beamline I24 at Diamond Light Source (DLS, UK) with a PILATUS2 6M hybrid pixel-array detector. Each diffraction image corresponded to an oscillation angle of 0.2° with diffraction observed to a maximum resolution of 2.05 Å. Data reduction was performed using *XDS* (Kabsch, 2010 ▸).

Data-collection and processing information is shown in Table 3 ▸.

### 2.4. Structure solution and refinement   

**Figure 1:**
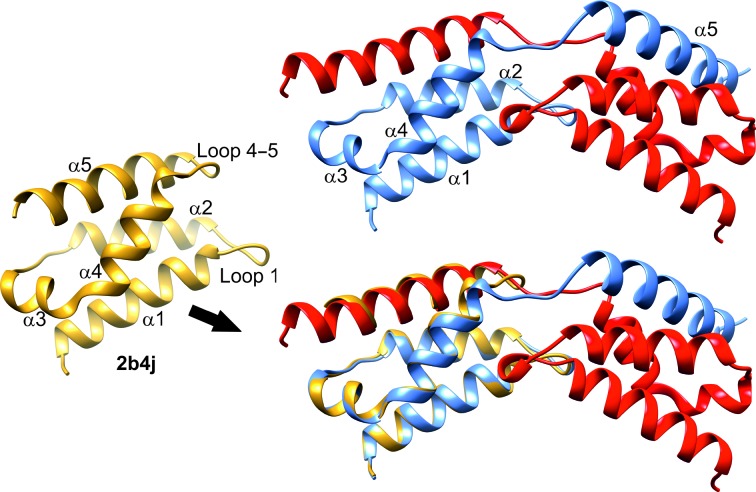
IBD domain-swapped dimer (chain G, red; chain H, blue) viewed perpendicular to the local twofold axis (top). The IBD domain from the human integrase complex (PDB entry 2b4j) is shown in yellow (left), together with its superposition on chain H (bottom). All helices superimpose well, with the only significant disruption in loop 4–5. Figures were prepared with *UCSF Chimera* (Pettersen *et al.*, 2004 ▸).

The crystals of IBD belonged to space group *P*2_1_, with unit-cell parameters *a* = 71.18, *b* = 54.81, *c* = 118.00 Å, β = 91.23°, and contained eight molecules per asymmetric unit, giving a Matthews coefficient and solvent content of 2.43 Å^3^ Da^−1^ and 49.48%, respectively (Matthews, 1977 ▸). Initial phase estimates were calculated using *Phaser* (McCoy *et al.*, 2007 ▸) from the *CCP*4 software suite (Winn *et al.*, 2011 ▸) with the IBD domain from PDB entry 2b4j (Cherepanov, Sun *et al.*, 2005 ▸) as a search model. The structure was refined using iterative cycles of *REFMAC*5 (Murshudov *et al.*, 2011 ▸) followed by manual rebuilding of the model using *Coot* (Emsley *et al.*, 2010 ▸). The electron density clearly showed all five α-helices, but the loop linking helices α4 and α5 (loop 4–5; residues 405–409) was less well defined and did not follow the expected path of the loop, instead leading to density belonging to an adjacent IBD molecule. As refinement progressed it became clear that helix α5 in each IBD domain actually belonged to a neighbouring IBD chain, and that there was a domain swap with concomitant reorganization of loop 4–5 (Fig. 1 ▸). The octamer in the asymmetric unit is composed of four domain-swapped dimers with good overall noncrystallographic symmetry (NCS), and eightfold NCS-averaged maps were used in initial model building, with eightfold NCS restraints applied in refinement (the r.m.s. fit between equivalent C^α^ atoms in the final refined model is 0.70 Å). Evidence of disorder, however, remained in loop 4–5, and helix α5 did not obey the NCS as faithfully as the rest of the domain. The two loop 4–5 regions in each domain-swapped dimer do not obey the local twofold NCS and, in particular, the two Phe406 side chains would clash with each other if both were in the same conformation. There are at least two conformations for loop 4–5, and inspection of each dimer in turn showed a majority population of one conformation in one subunit and of the other in the related subunit, leading to asymmetry in each dimer. The four dimers (chains AB, CD, EF and GH) were rebuilt with the asymmetric linkers, and refinement proceeded applying full eightfold NCS restraints to residues 345–403 (helices α1–α4) and, separately, to residues 408–431 (helices α5), with fourfold NCS restraints applied to residues 404–407 in chains A, C, F and G and chains B, D, E and H, respectively. Residual density remains around loop 4–5, indicating a degree of disorder. The N-terminal linker regions preceding the IBD in the expression construct, and 24 of the C-terminal residues in the IBD octamer were not visible in the electron density, so that the final model lacks 209 disordered residues that were shown to be present by mass spectrometry.

The final refined model has crystallographic *R*_work_ and *R*_free_ values of 18.2 and 23.6%, respectively. The quality of the structure was evaluated using *MolProbity* (Chen *et al.*, 2010 ▸), with a *MolProbity* score of 1.48, corresponding to the 97th percentile for structures at comparable resolution. Figures were prepared using *UCSF Chimera* (Pettersen *et al.*, 2004 ▸). Coordinates and structure factors have been deposited in the Protein Data Bank (http://www.rcsb.org) with accession code 5oym.

Data-reduction and refinement statistics are shown in Table 4 ▸.

## 3. Results and discussion   

The HIV integrase-binding domain (IBD) of LEDGF has been cloned, expressed and purified, and its crystal structure has been determined to 2.05 Å resolution. The electron-density map was of good quality for the eight crystallo­graphically independent polypeptide chains (A–H) in the asymmetric unit, allowing residues 345–429 to be built in all of them. The IBD domain consists of four long α-helices (α1, α2, α4 and α5) arranged as a helical bundle, with a fifth short helix, α3, linking α2 and α4 (Fig. 1 ▸). The remaining links between helices are made by two short loops: loop 1–2 and loop 4–5. The crystal structure of the IBD domain has previously been reported in complexes with human HIV integrase (Cherepanov, Ambrosio *et al.*, 2005 ▸; PDB entry 2b4j) and an HIV integrase homologue (Hare & Cherepanov, 2009 ▸; PDB entry 3f9k). The structure of the free IBD domain has also been solved in solution by NMR (Cherepanov, Sun *et al.*, 2005 ▸; PDB entry 1z9e). All of the published IBD structures have a single, very similar, compact domain, such as that of PDB entry 2b4j shown in Fig. 1 ▸, but the free IBD crystal structure reported here shows a domain-swapped dimer. Reorganization of loop 4–5 allows α5 of each IBD to cross over and occupy its normal location relative to the other domain (Fig. 1 ▸). A least-squares fit of the C^α^ atoms for residues 347–405 (helices α1–α4) and 410–427 (helix α5) of PDB entry 2b4j to residues 347–405 (helices α1–α4) of IBD chain G and 410–427 (helix α5) of IBD chain H gives an r.m.s deviation of 0.49 Å, showing that the packing of the swapped helix α5 closely matches its location in the monomeric 2b4j domain.

**Figure 2:**
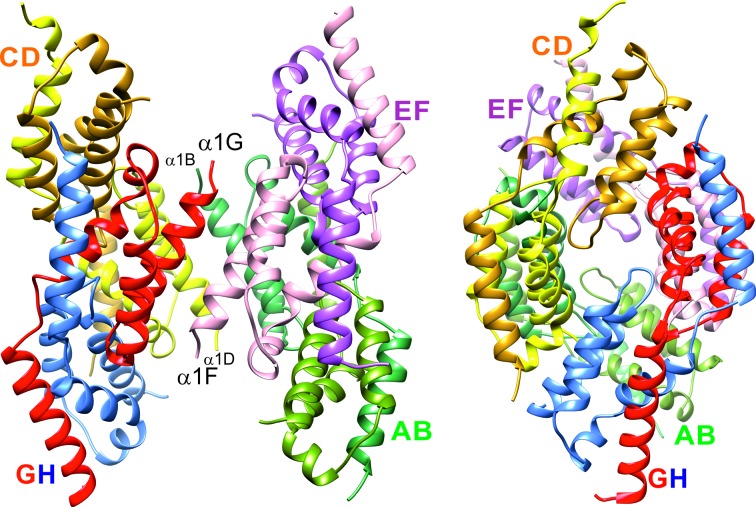
The IBD octamer viewed along two perpendicular twofold axes. Pairs of domain-swapped dimers assemble into tightly packed tetramers AB/EF and CD/GH (right). The two tetramers associate less strongly *via* contacts between the N-terminal helices of chains B, D, F and G.

In the crystal structure, four domain-swapped IDB dimers further assemble into octamers with 222 symmetry (Fig. 2 ▸). Interestingly, the local twofold axes of the IBD dimers do not pass through the centre of the octamer and are therefore not part of the point group, so that the octamer corresponds to a symmetric tetramer of dimers. Pairs of domain-swapped IBD dimers pack tightly together to form two equivalent tetramers: AB/EF and CD/GH. In the AB/EF tetramer interfaces α3B, α4B and the C-terminus of α5A pack against α1E and α2E, while the symmetry-related α3F, α4F and the C-terminus of α5E pack against α1A and α2A, with a similar arrangement in the CD/GH tetramer. The two tetramers associate more loosely *via* the N-terminal helices α1B, α1D, α1F and α1G of chains B, D, F and G, which are not buried in the tetramer interfaces, to form the octamer.

Domain swapping in proteins is not uncommon, and has been reviewed by Liu & Eisenberg (2002 ▸). An online database of domain-swapped structures (http://caps.ncbs.res.in/3dswap; Shameer *et al.*, 2010 ▸) shows 293 entries in the PDB. While domain swapping is sometimes functional, and pathological in the case of amyloid proteins, there are many cases where it is an artefact, frequently where a domain has been separated from the rest of a larger protein. These cases usually involve the N- or C-termini, and it has been suggested that this can occur under appropriate conditions for virtually any protein with an unconstrained terminus (Liu & Eisenberg, 2002 ▸; Bonjack-Shterengartz & Avnir, 2017 ▸; Gronenborn, 2009 ▸).

**Figure 3:**
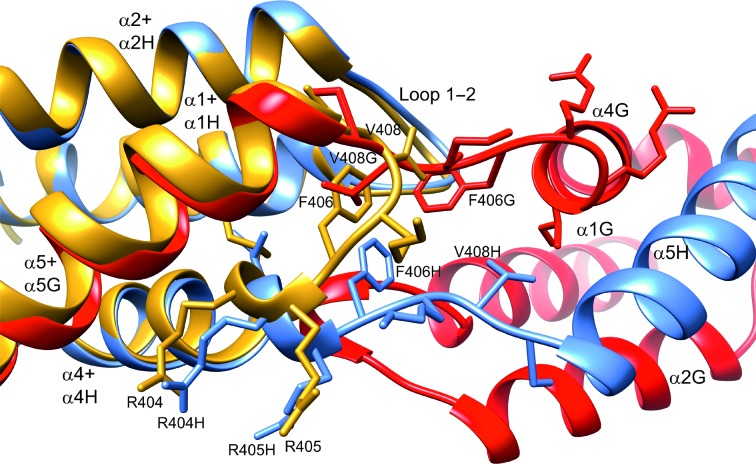
Close-up of the domain-exchange ‘hinge loop’ of the dimer (chain G, red; chain H, blue) viewed approximately along the local twofold axis, with the superimposed IBD domain from the human integrase complex (PDB entry 2b4j) shown in yellow. Residue and helix labels without chain identifiers correspond to PDB entry 2b4j. The side chain of Phe406 in the 2b4j monomer is replaced by Val408G of the opposing subunit in the dimer.

In the case of IBD, α5 does not appear to be unconstrained, but may be destabilized by removal of the domain from the rest of the LEDGF protein. The ‘hinge loop’ is the point of exchange in domain-swapped proteins and frequently forms either a β-strand or α-helix (Liu & Eisenberg, 2002 ▸). Loop 4–5 in IBD corresponds to the ‘hinge loop’, and it adopts an extended β-strand conformation for residues 405–409 in the domain-swapped dimer. In the monomeric forms, such as PDB entry 2b4j, it forms a type I β-turn at residues 406–409, and the only significant conformation change in the domain-swapped form is the conversion of Lys407 and Val408 from α-helical to β-strand φ and ψ angles (Fig. 3 ▸). In the monomeric form, the side chain of Phe406 packs against the end of the helical bundle, with Val408 lying on top at the surface of the loop. In the domain-swapped dimer, the phenyl ring of Phe406 is replaced by the side chain of Val408 from the other subunit, with its corresponding Phe406 phenyl ring packed behind it (Fig. 3 ▸). Steric crowding at the crossover point in the dimer prevents the two opposing Phe406 side chains from adopting the same conformation, with their rotamers differing principally in χ_2_, leading to a breakdown of twofold symmetry and the disorder that is observed in electron-density maps.

**Figure 4:**
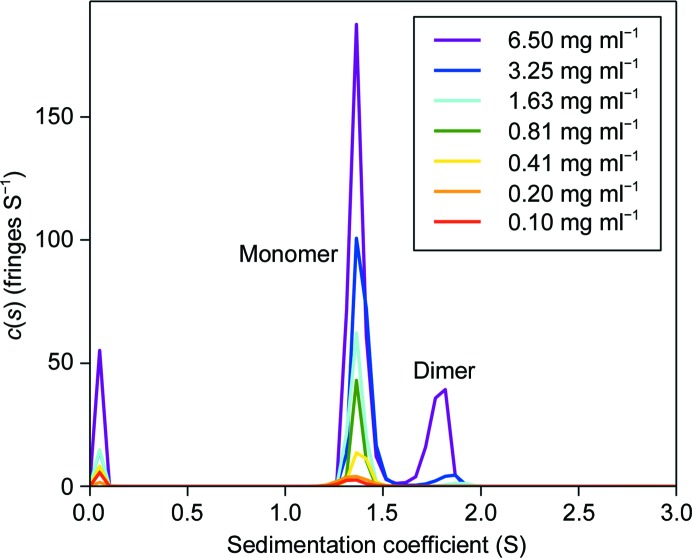
Sedimentation-coefficient distributions of IBD at a range of concentrations. There is clear evidence of dimer formation above 3 mg ml^−1^.

The observation of domain-swapped dimers in the crystal raises the question of whether the dimers are present in solution or are purely an artefact of crystallization. Analytical ultracentrifugation (AUC) of IBD solutions at a range of concentrations in a buffer similar to that used for crystallization showed the presence of dimers at concentrations greater than 3 mg ml^−1^. Fig. 4 ▸ shows sedimentation-coefficient distributions of IBD obtained from the interference data in AUC and, although the self-association is not saturated, implies a *K*_d_ for dimer formation of approximately 2 m*M*. It is clear that dimerization is a property of IBD at millimolar concentrations and is not induced by crystallization. There is no evidence, however, of the more loosely packed octamers in solution, and these may only exist in the crystal.

The IBD structure was determined as a target for structure-based drug design in a study aimed at inhibiting protein–protein interaction between LEDGF and HIV integrase as a strategy for antiviral treatment for AIDS. A single VH antibody domain was isolated that binds to LEGDF and blocks the binding of HIV integrase (Bao *et al.*, 2017 ▸; Tanaka & Rabbitts, 2010 ▸). In the integrase complex, the interface between the integrase and IBD is the surface formed by loops 1–2 and 4–5 (Cherepanov, Ambrosio *et al.*, 2005 ▸; PDB entry 2b4j). The VH domain also binds IBD, and the crystal structure of the VH–IBD complex shows the same binding site on IBD as the integrase (Bao *et al.*, 2017 ▸; PDB entry 5n88). The domain-swapped dimer in the crystal, however, does not present the same binding site as the monomer at loops 1–2 and 4–5, showing that it is not the intracellular form of IBD. Caution should therefore be used in the ‘divide-and-rule’ strategy that employs isolated domains of larger proteins as surrogates for structure-based design.

## Supplementary Material

PDB reference: HIV integrase-binding domain of lens epithelium-derived growth factor, 5oym

Supplementary Figure S1.. DOI: 10.1107/S2053230X18001553/pg5073sup1.pdf

## Figures and Tables

**Table 1:** Macromolecule-production information

Source organism	Human
DNA source	cDNA prepared with the ProtoScript II RT-PCR kit with RNA extracted from the T-cell lymphoma cell line VL3-3M2
Forward primer	ATTGCGGCCGCAATGGTTAAGAAAGTGGAGAAGAAGCGA
Reverse primer	ATAGAATTCTTATTCACCAACCAAAAACATATT
Cloning vector	pRK172
Expression vector	pRK172
Expression host (crystallization)	*E. coli* strain B834 (DE3)
Expression host (analytical ultracentrifugation)	*E. coli* strain C41 (DE3)
Complete amino-acid sequence of the construct produced	MVKKVEKKRHHHHHHGSENLYFQGGSMGSGGGGSGGGGSGGGGAAAMETSMDSRLQRIHAEIKNSLKIDNLDVNRCIEALDELASLQVTMQQAQKHTEMITTLKKIRRFKVSQVIMEKSTMLYNKFKNMFLVGE

**Table 2:** Crystallization

Method	Vapour diffusion, sitting drop
Plate type	96-well 2-drop MRC crystallization plates
Temperature (K)	294
Protein concentration (mg ml^−1^)	6.5
Buffer composition of protein solution	20 m*M* Tris pH 7.5, 200 m*M* NaCl, 1 m*M* TCEP
Composition of reservoir solution	0.2 *M* sodium fluoride, 0.1 *M* bis-tris propane pH 8.5, 20%(*w*/*v*) PEG 3350
Volume and ratio of drop	100 nl, 1:1
Volume of reservoir (µl)	95

**Table 3:** Data collection and processing Values in parentheses are for the outer shell.

Diffraction source	Beamline I24, DLS
Wavelength (Å)	0.9778
Temperature (K)	100
Detector	PILATUS2 6M
Crystal-to-detector distance (mm)	390
Rotation range per image (°)	0.2
Total rotation range (°)	125.8
Exposure time per image (s)	0.2
Space group	*P*2_1_
*a*, *b*, *c* (Å)	71.18, 54.81, 118.00
α, β, γ (°)	90, 91.23, 90
Mosaicity (°)	0.103
Resolution range (Å)	54.81–2.05 (2.16–2.05)
Total No. of reflections	132649 (19728)
No. of unique reflections	55617 (8059)
Completeness (%)	97.1 (97.0)
Multiplicity	2.4 (2.4)
〈*I*/σ(*I*)〉	8.6 (1.7)
CC_1/2_	0.997 (0.510)
*R*_r.i.m._	0.097 (0.796)
Overall *B* factor from Wilson plot (Å^2^)	28.8

**Table 4:** Structure refinement Values in parentheses are for the outer shell.

Resolution range (Å)	14.98–2.05 (2.102–2.050)
Completeness (%)	96.3
σ Cutoff	None
No. of reflections, working set	52601 (3819)
No. of reflections, test set	2800 (200)
Final *R*_cryst_	0.182 (0.297)
Final *R*_free_	0.236 (0.322)
No. of non-H atoms	
Protein	5606
Solvent	445
Total	6129
R.m.s. deviations	
Bonds (Å)	0.017
Angles (°)	1.88
Average *B* factors (Å^2^)	
Protein	38.2
Water	40.0
Ramachandran plot	
Favoured regions (%)	97.5
Additionally allowed (%)	2.5
