# Supplementary material for: Cloning, purification and structure determination of the HIV integrase-binding domain of lens epithelium-derived growth factor
Source: Acta Crystallogr F Struct Biol Commun. 2018 Feb 26;74(Pt 3):143–9. doi: 10.1107/S2053230X18001553 (PMC5947699; doi:10.1107/S2053230X18001553)
Supplement: Supplementary file 1 [file f-74-00143-sup1.pdf]

Acta Cryst F
STRUCTURAL BIOLOGY
COMMUNICATIONS

Volume 74 (2018)

Supporting information for article:

Cloning, purification and structure determination of the HIV integrase-binding domain of lens epithelium-derived growth factor

Clare Hannon, Abimael Cruz-Migoni, Olga Platonova, Robin L. Owen, Joanne E. Nettleship, Ami Miller, Stephen B. Carr, Gemma Harris, Terence H. Rabbitts and Simon E. V. Phillips

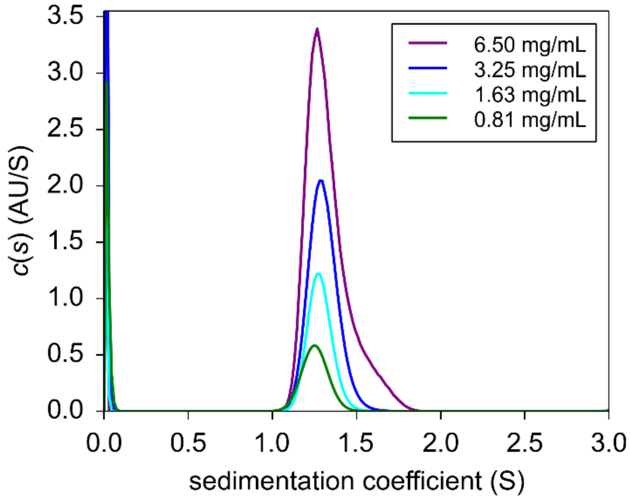

Figure S1

Sedimentation coefficient distributions of IBD obtained from the AUC absorbance data. Only distributions obtained from absorbance measurements of samples in the linear range of the detector are shown. The absorbance data, being collected at a lower radial resolution, is less able to distinguish the dimer species from the monomer, however, the sedimentation coefficient of the peak clearly shifts progressively to a higher value with increasing IBD concentration and the dimer begins to appear as a shoulder on the right-hand side of the peak.
